# Safety and effectiveness of high-dose vitamin C in patients with COVID-19: a randomized open-label clinical trial

**DOI:** 10.1186/s40001-021-00490-1

**Published:** 2021-02-11

**Authors:** Saeidreza JamaliMoghadamSiahkali, Besharat Zarezade, Sogol Koolaji, SeyedAhmad SeyedAlinaghi, Abolfazl Zendehdel, Mohammad Tabarestani, Ehsan Sekhavati Moghadam, Ladan Abbasian, Seyed Ali Dehghan Manshadi, Mohamadreza Salehi, Malihe Hasannezhad, Sara Ghaderkhani, Mohsen Meidani, Faeze Salahshour, Fatemeh Jafari, Navid Manafi, Fereshteh Ghiasvand

**Affiliations:** 1 Department of Infectious Diseases, Ziayian Hospital, Tehran University of Medical Sciences, Tehran, Iran; 2 Department of Infectious Diseases, Imam Khomeini Hospital Complex, Tehran University of Medical Sciences, Tehran, Iran; 3 Department of Cardiology, Firoozgar Hospital, Iran University of Medical Sciences, Tehran, Iran; 4 Iranian Research Center for HIV/AIDS, Iranian Institute for Reduction of High Risk Behaviors, Tehran University of Medical Sciences, Tehran, Iran; 5 Geriatric Department, Ziayian Hospital, Tehran University of Medical Sciences, Tehran, Iran; 6 Students Research Committee, School of Medicine, Mazandaran University of Medical Sciences, Sari, Iran; 7 Department of Cardiology, Ziayian Hospital, Tehran University of Medical Sciences, Tehran, Iran; 8 Department of Radiology, Imam Khomeini Hospital Complex, Tehran University of Medical Sciences, Tehran, Iran; 9 Max Rady College of Medicine, University of Manitoba, Winnipeg, Canada; 10 Liver Transplantation Research Center, Department of Infectious Diseases, Imam Khomeini Hospital Complex, Tehran University of Medical Sciences, Keshavarz Boulevard, Tehran, Iran

**Keywords:** COVID-19, SARS-COV-2, 2019-nCoV, Vitamin C, Pneumonia, Hydroxychloroquine, Lopinavir, Ritonavir

## Abstract

**Background:**

Vitamin C is an essential water-soluble nutrient that functions as a key antioxidant and has been proven to be effective for boosting immunity. In this study, we aimed to assess the efficacy of adding high-dose intravenous vitamin C (HDIVC) to the regimens for patients with severe COVID-19 disease.

**Methods:**

An open-label, randomized, and controlled trial was conducted on patients with severe COVID-19 infection. The case and control treatment groups each consisted of 30 patients. The control group received lopinavir/ritonavir and hydroxychloroquine and the case group received HDIVC (6 g daily) added to the same regimen.

**Results:**

There were no statistically significant differences between two groups with respect to age and gender, laboratory results, and underlying diseases. The mean body temperature was significantly lower in the case group on the 3rd day of hospitalization (*p* = 0.001). Peripheral capillary oxygen saturations (SpO_2_) measured at the 3rd day of hospitalization was also higher in the case group receiving HDIVC (*p* = 0.014). The median length of hospitalization in the case group was significantly longer than the control group (8.5 days vs. 6.5 days) (*p* = 0.028). There was no significant difference in SpO_2_ levels at discharge time, the length of intensive care unit (ICU) stay, and mortality between the two groups.

**Conclusions:**

We did not find significantly better outcomes in the group who were treated with HDIVC in addition to the main treatment regimen at discharge.

*Trial registration* irct.ir (IRCT20200411047025N1), April 14, 2020

## Background

The coronavirus disease 2019 (COVID-19) pandemic which started at late 2019 and spread the world outrageously is caused by infection with Severe Acute Respiratory Syndrome Coronavirus 2 (SARS-CoV-2), a member of the coronaviridae family. By January 2021, more than two million lives have been sacrificed by this disease, even more deaths are expected unless proper management does not take into place soon, in terms of prevention, transmission control, and treatment. Ascorbic acid or ascorbate (vitamin C) is an essential water-soluble nutrient that functions as a key antioxidant and is involved in the synthesis of collagen and neurotransmitters, and affects wound healing, energy metabolism, nervous system function, and immune cell health [1–3]. The serum level of this vitamin has been correlated with its effect on the endothelial function [4], cellular immune function [5], anti-oxidative capacity, neutrophil function [6], and even for treatment of cancer and pancreatitis [7, 8]. Intravenous (IV) administration increases the plasma ascorbate concentrations more than oral supplementation (30 mM vs. 0.2 mM, respectively) [9, 10].

The evidence behind theoretical possible effect of vitamin C against COVID-19 is promising [11]. In a clinical study of the role of ascorbic acid against Epstein–Barr virus (EBV) infection showed the EBV IgG and IgM antibody levels reduced during IV vitamin C therapy [12]. Also in a case report of enterovirus/rhinovirus-induced acute respiratory distress syndrome (ARDS) in 2017, infusion of high-dose IV vitamin C (HDIVC) was associated with rapid resolution of lung injury [13]. The impact of vitamin C administration on alleviating lung injury has also been investigated and supported in other studies [14]. There are other studies expressing the positive effect of IV vitamin C in patients with severe sepsis [15–17]. A meta-analysis also reported the impact of vitamin C on decreasing the duration of intensive care unit (ICU) admission and mechanical ventilation care in patients with ARDS [18–20].

Given the positive effect of IV vitamin C for viral-induced ARDS and its role for enhancing the function of immune system, we aimed to investigate the correlation of the HDIVC administration with improvement of 2019-nCoV-induced ARDS. There is lack of data and clinical trials that studied this correlation recently.

## Materials and methods

### Participants

**Fig. 1:**
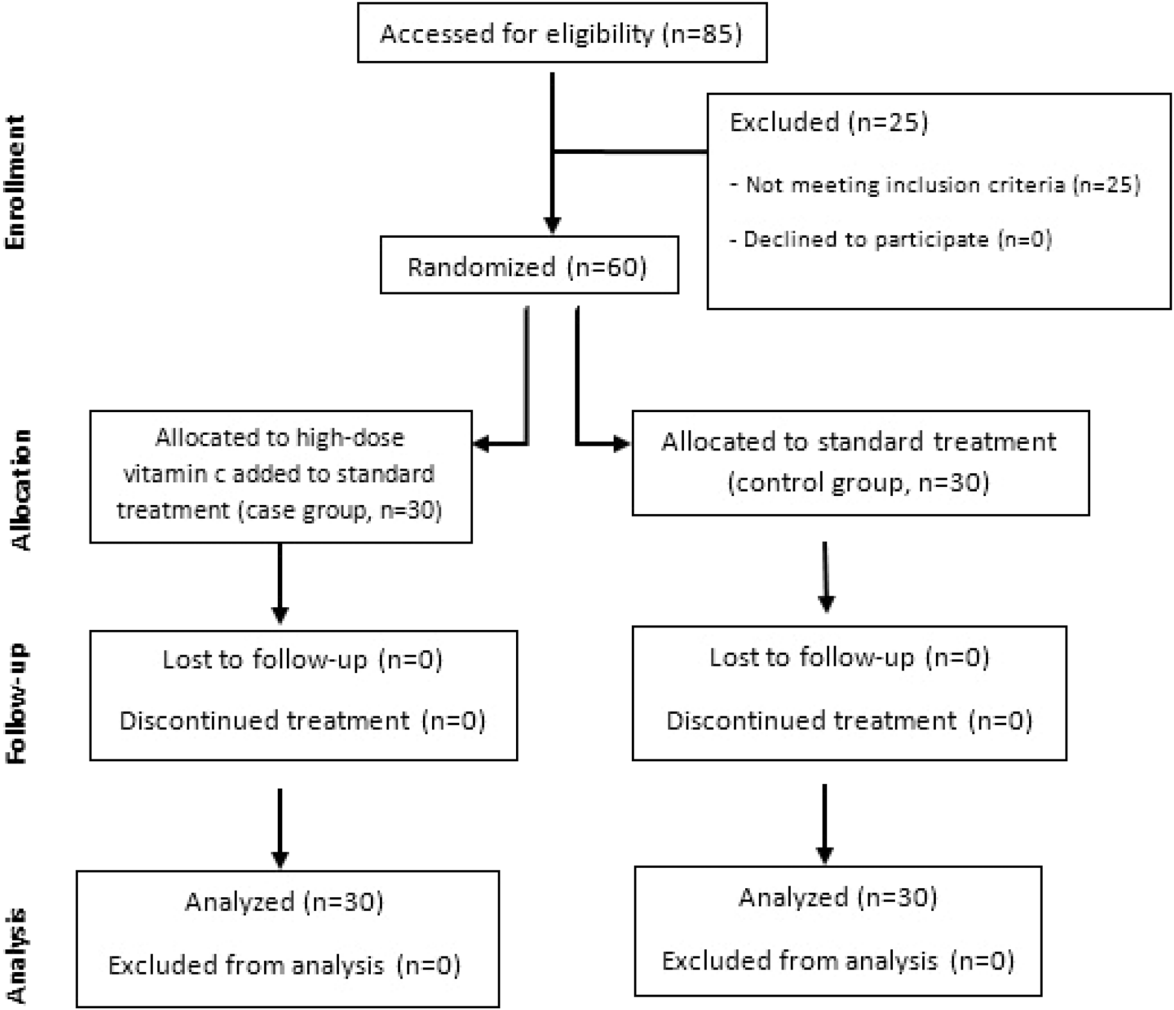
Randomization and treatment assignment

Between April and May 2020, 85 patients with compelling clinical symptoms for diagnosis of COVID-19 were admitted to Ziaeian Hospital, Tehran, Iran. Based on the eligibility criteria (Fig. 1), 25 patients were excluded and 60 patients were included in the study. The inclusion criteria were age older than 18 years, positive COVID-19 polymerase chain reaction (PCR) test or COVID-19 suspicion based on clinical findings (mainly fever, dyspnea, dry cough), imaging findings of COVID-19 on spiral chest computer tomography (CT) or high-resolution CT (HRCT) imagings validated by a trained radiologist, clinical manifestations of ARDS or myocarditis, and oxygen saturation lower than 93% from admission or after 48 h from the first COVID-19 treatment. The exclusion criteria were receiving anti-retroviral therapy or immune system booster medications in the last 3 months, no proven and confirmed COVID-19 disease based on the inclusion criteria, patients with glucose-6-phosphate dehydrogenase (G6PD) deficiency, patients with end-stage renal disease (ESRD), and pregnancy.

### Study arms and treatment plans

The patients were divided into two subgroups equally by block randomization; the case group included 30 patients receiving 1.5 g vitamin C IV every 6 h for 5 days and the control group included 30 patients who did not receive vitamin C. All of the participants were also treated with oral lopinavir/ritonavir (Kaletra, Abbott Laboratories) 400/100 mg twice daily and single stat dose of oral hydroxychloroquine (400 mg) on the first day of hospitalization according to the Iranian COVID-19 treatment protocol at time of this study (it should be noted that based on the vast number of studies for COVID-19, hydroxychloroquine is not considered as mainstay in the protocol for COVID-19 in Iran anymore). On the first day of hospitalization, laboratory studies including complete blood count (CBC), C-reactive protein (CRP), and erythrocyte sedimentation rate (ESR) were obtained. Patients were assessed by daily measurements of core body temperature, respiratory rate (RR), heart rate (HR), and peripheral capillary oxygen saturations (SpO_2_). The treatment subsided whenever any kind of drug side effects appeared. Some of the patients deteriorated during the admission and received corticosteroid (methylprednisolone 125 mg daily for 3 days). Patients were discharged when they achieved a stable SpO_2_ > 92%, no evidence of respiratory distress was remaining, and were afebrile for at least 3 consecutive days.

### Ethical considerations

In accordance to the Declaration of Helsinki, written informed consent was obtained from all participants before initiation of the study. The patients were assured that declining to participate in the study or leaving the study at any point would not affect the quality of their treatment and that they would thereafter receive the standard care. The study protocol was approved by the institutional review board (IRB) of Tehran University of Medical Sciences (TUMS) (IR.TUMS.VCR.REC.1399.078).

### Measurements and statistical analysis

In this open-label and nonblinded study, distribution of age, gender, initial clinical symptoms, and vital signs of the first day of admission were compared between the two groups. The vital signs including body temperature, RR, HR and SpO_2_ were also compared on the 3rd and last day of treatment between the two groups as an outcome measure. Differences in duration of hospitalization, number of patients whose condition deteriorated and needed ICU admission, length of ICU admission, and difference between mortality rates were measured. The primary endpoints in this trial were a decrease in mortality, duration of hospitalization, and need for ICU admission. Secondary endpoints were determined as improvements in SpO_2_ and vital signs as well as the general wellbeing of the patient. Severity score was calculated based on the scoring system suggested by Altschul et al. for prediction of inpatient mortality in COVID-19 patients [21]. Based on this scoring system, the higher the score of the patient, the higher the odds of inpatient mortality.

Sample size calculation was performed for non-inferiority tests of difference between two group proportions. We assumed an effectiveness of 65% for the intervention group and effectiveness of 50% for the control group. We also assumed a margin of non-inferiority of at least 10% between the two groups. The power of the study was determined as 90% (G ∗ Power, Erdfelder, Faul, and Buchner, 1996).

Data was analyzed using SPSS software (IBM Corp. Released 2013. IBM SPSS Statistics for Windows, Version 22.0. Armonk, NY: IBM Corp.). Quantitative variables are reported by mean and standard deviation (SD) and qualitative variables are reported using frequency and percentage. Because of the normal distribution of our data via Shapiro–Wilk test, the independent *t*-test was used to assess the means differences and a mixed-design analysis of variance model (ANOVA) was performed to evaluate the effect of time on body temperature. Chi-square and Fisher’s exact tests were used to assess the statistical relationships between categorical variables. The level of significance was set as *p*-value < 0.05 for all analyses.

## Results

### Baseline characteristics

Demographic characteristics, underlying diseases, and clinical and laboratory findings are presented in Table 1. Male-to-female ratio was 1:1. There were no statistically significant differences between two groups considering age and gender, laboratory results and underlying diseases (*p* > 0.05). All clinical findings except for fever (23.33% vs. 63.33% in case and control groups, respectively, *p* = 0.002) and myalgia (13.33% vs. 60.0% in case and control groups, respectively, *p* < 0.001) were not significantly different between the two groups.

**Table 1:** Demographic characteristics, underlying diseases, clinical and laboratory findings, and outcomes

	Group		*p*
	Case (*n* = 30)	Control (*n* = 30)	
Age (year), mean (SD)	57.53 (18.27)	61 (15.90)	0.436
Sex, *n* (%)			
Female	15 (50.00%)	15 (50.00%)	> 0.9
Male	15 (50.00%)	15 (50.00%)	
Hypertension, *n* (%)	15 (50.00%)	10 (33.33%)	0.190
Diabetes mellitus, *n* (%)	12 (40.00%)	11 (36.67%)	0.791
Ischemic heart disease, *n* (%)	4 (13.33%)	7 (23.33%)	0.506
COPD, *n* (%)	3 (10.00%)	3 (10.00%)	> 0.9
Thyroid disease, *n* (%)	2 (6.67%)	3 (10.00%)	> 0.9
Fever, *n* (%)	7 (23.33%)	19 (63.33%)	**0.002**
Chill, *n* (%)	7 (23.33%)	9 (30.00%)	0.559
Dyspnea, *n* (%)	25 (83.33%)	21 (70.00%)	0.222
Myalgia, *n* (%)	4 (13.33%)	18 (60.00%)	**< 0.001**
Weakness, *n* (%)	2 (6.67%)	4 (13.33%)	0.671
Cough, *n* (%)	26 (86.67%)	23 (76.67%)	0.506
Sputum, *n* (%)	1 (3.33%)	4 (13.33%)	0.353
Headache, *n* (%)	3 (10.00%)	8 (26.67%)	0.181
Vomit, *n* (%)	4 (13.33%)	2 (6.67%)	0.671
Chest pain, *n* (%)	2 (6.67%)	5 (16.67%)	0.424
Hemoptysis, *n* (%)	0 (0%)	3 (10.00%)	0.237
Positive PCR, *n* (%)	22 (73.33%)	30 (100.00%)	> 0.9
Negative PCR, *n* (%)	8 (26.66%)	0 (00.00%)	
WBC count (× 10^3^/µl), mean (SD)	6.60 (3.65)	6.43 (3.69)	0.861
Lymphocyte (count/µl), mean (SD)	1082.68 (582.17)	1042.52 (590.81)	0.792
HB (g/dl), mean (SD)	13.35 (2.29)	12.65 (2.06)	0.218
PLT (× 10^3^/µl), mean (SD)	194.20 (83.75)	203.30 (74.64)	0.658
AST (u/l), mean (SD)	35.93 (15.92)	33.93 (13.96)	0.607
ALT (u/l), mean (SD)	31.73 (8.57)	34.43 (9.69)	0.258
LDH (u/l), mean (SD)	619.20 (212.10)	599.67 (197.93)	0.714
CRP (mg/dl), mean (SD)	41.30 (28.86)	58.13 (52.80)	0.132
ESR (mm/hour), mean (SD)	60.00 (30.71)	66.03 (30.45)	0.448
Body temperature upon admission(°C), mean (SD)	37.03 (0.80)	37.93 (0.92)	**< 0.001**
3rd day temperature (°C), mean (SD)	36.73 (0.36)	37.24 (0.69)	**0.001**
Body temperature at discharge (°C), mean (SD)	36.76 (0.47)	36.85 (0.46)	0.454
SPO_2_ upon admission (%), median (IQR)	86.0 (82.0–88.0)	87.5 (85.0–88.0)	0.148
3rd day SPO_2_ (%), median (IQR)	90.5 (88.0–92.0)	88.0 (80.0–91.0)	**0.014**
SPO_2_ at discharge (%), median (IQR)	93.5 (91.0–95.0)	92.5 (92.0–94.0)	0.406
ICU length of stay (day), median (IQR)	5.50 (5.0–10.0)	5.0 (5.0–7.0)	0.381
Hospital length of stay (day), median (IQR)	8.50 (7.0–12.0)	6.50(4.0–12.0)	**0.028**
Expire, *n* (%)	3 (10.00%)	3 (10.00%)	> 0.9
Intubation, *n* (%)	5 (16.67%)	4 (13.33%)	> 0.9
Corticosteroid treatment, *n* (%)	8 (26.66%)	7 (23.33%)	0.77
Severity score, mean (/10) (SD)	3.57 (1.357)	3.40 (1.476)	0.651

*SD* standard deviation, *n* count, *IQR* interquartile range (25–75%), *COPD* chronic obstructive pulmonary disease, *WBC* white blood cell, *HB* hemoglobin, *PLT* platelet, *AST* aspartate transaminase, *ALT* alanine transaminase, *LDH* lactate dehydrogenase, *CRP* C-reactive protein, *ESR* erythrocyte sedimentation rate, *SPO*_*2*_ oxygen saturation, *ICU* intensive care unit, *PCR* polymerase chain reaction

### Outcomes

**Fig. 2:**
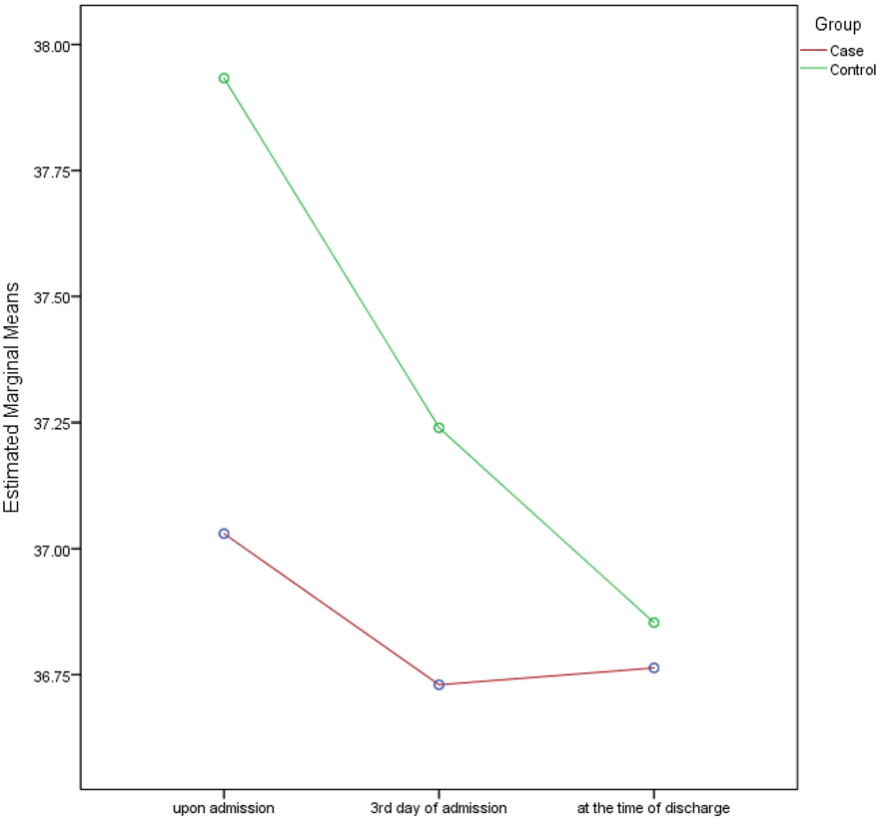
Body temperature means through time in the case and control groups

There was no significant difference in body temperature at the time of discharge between the two groups (*p* > 0.05) (Table 1). The mean body temperatures upon admission and on the 3rd day of admission were significantly higher in the control group (*p* = 0.001). The mixed-design analysis of variance model (ANOVA) performed to evaluate the effect of time on body temperature at the time of admission, on the 3rd day of admission and discharge, showed a significant effect of time on body temperature (Wilk’s Lambda = 0.589, *F* (2,57) = 19.879, *p* < 0.001) (Fig. 2). Post hoc comparison indicated a significant difference between body temperatures at time of admission, discharge, and on the 3rd day of hospitalization (*p* < 0.001).

SpO_2_ at admission and discharge were not significantly different between the two groups (*p* > 0.05) (Table 1). SpO_2_ on the 3rd day of admission was higher in the case group compared to the control group (median, 90.5% vs. 88.0%, respectively, *p* = 0.014) (Table 1). A non-parametric Friedman test of difference among repeated measures of SpO_2_ was conducted and there was a significant difference in mean ranks in both groups with the oxygen saturation increasing significantly in both groups (*p* < 0.001). The lowest SpO_2_ in the case group was 60% at admission who was discharged with 92%, and in the control group one patient had SpO_2_ of 60% who was discharged with SpO_2_ of 94%. Except these two cases, no patient had oxygen saturation below 70%. The case group had a median length of admission in the hospital of 8.5 (range 7.0–12.0) days which was significantly longer than the control group with a median length of admission of 6.5 (range 4.0–12.0) days. There was no significant difference in the length of ICU stay between the two groups (*p* > 0.05, Table 1). There was a non-significant higher rate of intubation in the case group (*p* > 0.05) (Table 1). Mortality rate was equal in both groups (three cases in each group, *p* > 0.05). During treatment with HDIVC, none of the patients experienced adverse events such as headache, nausea, bloating, or abdominal discomfort.

**Fig. 3:**
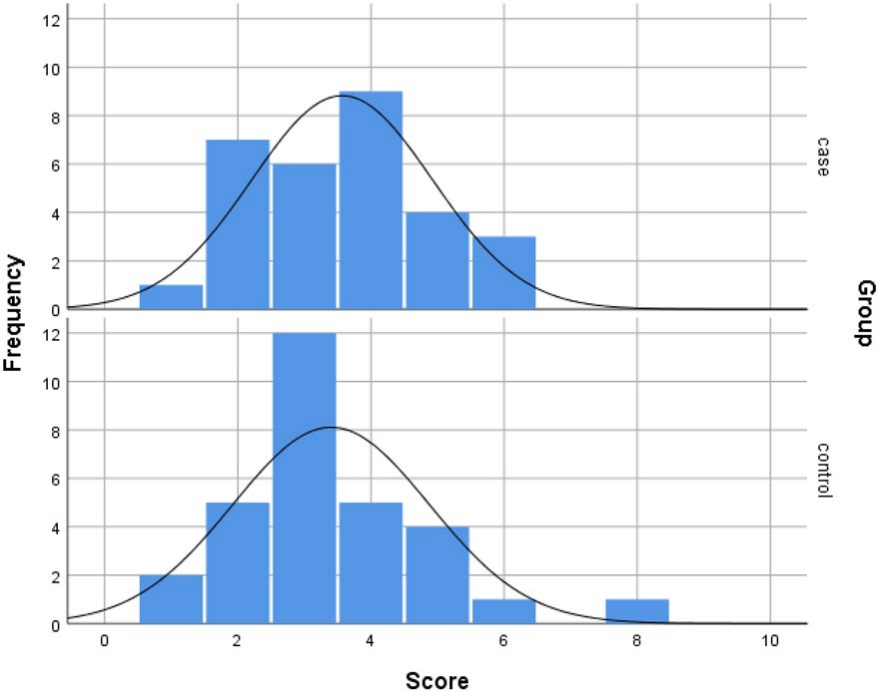
Distribution of severity scores of patients in each group

Figure 3 depicts the distribution of patients in both groups by their severity scores. It can be seen that despite minor differences between two groups, the diagram follows normal distribution and the difference between two groups is statistically insignificant (*p* = 0.651).

## Discussion

Until the time of this study, no definite treatment option has been suggested and cleared for COVID-19. While this pandemic is still responsible for death of above two million people and infection of many more, search for better treatment options should never be delayed [22, 23]. Vitamin C is an essential water-soluble nutrient that has important roles in our body, especially in immune cell functions [1, 2, 4]. Studies report that vitamin C can be effective in treatment of bacterial and viral infection [24–26]. These studies showed vitamin C weakly inhibits the multiplication of viruses such as influenza type A, Herpes simplex virus type 1 (HSV-1) and poliovirus type 1. A clinical study showed the effect of IV vitamin C therapy on reduction of IgG and IgM antibody levels in EBV infection [12]. There is also a report of a case of enterovirus/rhinovirus-induced ARDS where the infusion of HDIVC was associated with rapid resolution of lung injury [13].

Some studies showed that serum vitamin C levels may plummet in some patients especially in the critically ill during the course of infection [27, 28]; and vitamin C deficiency may contribute to organ injury and immune dysfunction which leads to the assumption that high doses of vitamin C might improve clinical outcomes of critically ill patients [27]. There is also some evidence that shows vitamin C may reduce patients’ susceptibility to lower respiratory tract infections such as pneumonia and it may have a protective role in lung infections, but further studies need to evaluate the efficacy of treatment with vitamin C in severe viral respiratory tract infections [27–31].

Meta-analyses demonstrated that the use of intravenous vitamin C as a therapy for sepsis and ARDS has benefits such as a lower rate of vasopressor requirements, shorter duration of both mechanical ventilation and admission in the ICUs; along with a shorter hospital admission in critically ill patients [19, 32–34]. Lin et al. found that administration of more than 50 mg/kg daily vitamin C had a significant effect in reduction of mortality rate in patients with severe sepsis. They concluded that a better survival rate correlated with administration of high doses of vitamin C [35]. Fowler et al. reported in their randomized, double-blind, placebo-controlled, multicenter trial that high doses of vitamin C did not significantly improve organ dysfunction scores in patients with severe sepsis or ARDS but in three secondary outcomes, use of vitamin C was associated with a significantly lower risk of mortality on the 28th day after diagnosis of the infection (29.8% vs. 46.3%), a higher number of ventilator-free days (13.1 vs. 10.6 days) and a higher number of ICU-free days (10.7 vs. 7.7 days) [36].

All these findings emphasize possible beneficial effects of vitamin C as a treatment for COVID-19. Here, we conducted a randomized clinical trial with 60 patients in two groups. Thirty patients were treated with 1.5 g of IV vitamin C, every 6 h for 5 days in addition to the main treatment regimen (case group), whereas the other 30 patients were treated only with the standard regimen. Demographic characteristics, underlying diseases, and clinical and laboratory findings were not significantly different between the two groups. Fever and myalgia were significantly more frequent in the control group but, other clinical findings were not notably different. SpO_2_ was improved in all patients. There is a similar report of SpO_2_ improvement associated with treatment with HDIVC (doses range from 2 to 10 g per day in 8–10-h IV infusions) in 50 moderate-to-severe COVID-19 patients. They also reported that all patients were cured and discharged [37]. However, the absence of a control group weakened the conclusions based on this report.

In the present study, there was no significant difference in oxygen SpO_2_ levels between the two groups at discharge, but the median of SpO_2_ levels were significantly higher in the case group on the 3rd day of admission. The mean body temperature significantly decreased during the admission in both groups and there was no significant difference between two groups regarding the core body temperature at discharge but, on the 3rd day of treatment, the mean of patients’ body temperature was significantly lower in the case group. Length of stay in the hospital had a median of 8.5 days and it was unexpectedly higher in the case group (8.5 vs. 6.5, *p* = 0.028). Other outcomes including number of deaths, number of intubations and duration of ICU admission were not significantly different between two groups. We did not find any side effects in the patients. Other studies also reported good tolerance of HDIVC in their trials [38].

There are not enough data and clinical trials that have evaluated the correlation between HDIVC treatment in COVID-19 patients with ARDS and improvement of their status, but there are several ongoing studies that aim to investigate the impact of high-dose vitamin C on COVID-19 patients (details of ongoing studies are presented in Table 2). Investigators in these studies will assess primary outcomes such as 50% reduction in symptoms score in 28 days, incidence of adverse effects (including severe adverse reactions), time to clinical improvement (TTIC), TTIC of National Early Warning Score 2 (NEWS2), number of hospital admission days, the rate of decline in lung infection rate, in-hospital mortality rates and number of ventilator-free days. The findings of these studies will be valuable and we hope to see promising results in their studies.

**Table 2:** Identifier and details of studies which investigated the advantages of high-dose vitamin C in patients with COVID-19

Identifier	Study type	Estimated enrollment	Allocation	Masking	Arms	Primary outcome measures
NCT04401150	CT	800	R	Q	Arm 1: Vitamin C: 50 mg/kg of weight administered intravenously every 6 h for 96 h (16 doses) Arm 2: Normal saline (0.9% NaCl) or dextrose 5% in water (D5W) in a volume to match the vitamin C	Death or persistent organ dysfunction
NCT04342728^*^	CT	520	R	O/L	Arm 1: 8000 mg of ascorbic acid (daily with food) Arm 2: 50 mg of zinc gluconate (daily) Arm 3: 8000 mg of ascorbic acid and 50 mg of Zinc gluconate	Symptom Reduction in 28 days
NCT04357782	CT	20	N/R	O/L	Arm 1: 50 mg/kg l-ascorbic acid (every 6 h for 4 days) in the group with mild deoxygenation Arm 2: 50 mg/kg l-ascorbic acid(every 6 h for 4 days) in the group with sever deoxygenation	1. Incidence of adverse events 2. Incidence of serious adverse reactions 3. Incidence of adverse reactions
NCT04323514	O	500	N/A	O/L	10 g of vitamin C intravenously in addition to conventional therapy	In-hospital mortality
NCT04344184^*^	CT	200	R	Q	Arm 1: 100 mg/kg intravenous vitamin C infusion (every 8 h 3 days) Arm 2: Dextrose 5% Water	Ventilator-free days
NCT04264533^*^	CT	140	R	T	Arm 1: 50 ml injection: 12 g vitamin C + Water (every 12 h for 7 days) Arm 2: 50 ml of sterile water (every 12 h for 7 days)	Ventilation-free days
IRCT20190917044805N2^**^	CT	60	R	D	Arm 1: 200 ml volume including 12,000 mg of vitamin C in dextrose 5% for 4 days Arm 2: 200 ml of Distilled water in dextrose 5%	1. Time to clinical improvement (TTIC) 2. Time to clinical improvement (TTIC) of NEWS2 (National Early Warning Score 2)
IRCT20200324046850N5^**^	CT	40	R	D	Arm 1: Main regime + 500 mg of vitamin C Arm 2: Main regime + Placebo of vitamin C	Number of hospital admission days
IRCT20151228025732N52^**^	CT	30	R	O/L	Arm 1: 2000 mg of vitamin C every 6 h for 7 days + main regime Arm 2: Only the main regime	1. The rate of decline in lung infection rate 2. Number of breaths per minute 3. The course of the disease 4. Heart rate

*CT* clinical trial, *O* observational study, *R* randomized, *N/A* not available, *N/R* non-randomized, *T* triple blinded, *Q* quadruple blinded, *D* double blinded, *O/L* open label

*ClinicalTrials.gov

**irct.ir

It is worthy to note that some studies have used higher doses of vitamin C in their patients [39, 40]. The highest dose was used in the work of Zhang et al. who have used 24 g daily in the form of 12 g of vitamin C/50 ml every 12 h for 7 days at a rate of 12 ml/h. Although they did not find superior results in terms of improving invasive mechanical ventilation-free days in 28 days (IMVFD28), but have found improving PaO2/FiO2 and hence benefiting oxygenation for critically ill patients [39]. Marik et al. also suggested MATH + protocol for COVID-19 patients, including methylprednisolone, ascorbic acid, thiamine, heparin, and supplemental oxygen. They suggest using vitamin C 3 g IV q 6 hourly for at least 7 days or until transferred out of ICU [40]. There are also other large randomized controlled trials (RCTs) using different doses of vitamin C. The largest RCT is the Lessening Organ Dysfunction with Vitamin C-COVID (LOVIT-COVID) trial in Canada assessing efficacy of 50 mg/kg every 6 h vitamin C, i.e., equivalent to 15 g/day for a 75 kg person (NCT04401150). Another large study is underway in Italy recruiting 500 patients in whom use of 10 g vitamin C is being tested. [NCT04323514] Although the results of these trials will also help our understanding of the preferred dose of vitamin C in COVID-19 patients, the available results point to the fact that higher doses can also be safe and effective (compared to the six g daily dose used in this study).

Another important topic is the length of vitamin C administration. Some studies suggest better outcome by administering vitamin C for longer duration, i.e., for at least 7 days [40]. However, a recent meta-analysis and systematic review of 17 studies reporting the effect of vitamin C supplementation in COVID-19 concluded that the best duration for administering vitamin C is over 3–4 days, with lower efficacy if used less than 3 days or more than 5 days [41].

Despite practicing proper randomization, certain factors were different between two groups (higher rate of myalgia and mean body temperature in control group), which can be overcome by having a larger patient population. Although by clinical judgment, one can assume that myalgia does not have much role in the prognosis of patients and given its subjectivity, it is of lower significance compared to other important prognostic factors. Also for body temperature, in this study more emphasis was put on the progression of patient symptoms. By viewing the results at different days of hospitalization, one can see that at discharge, both groups had values below the threshold of considering as fever. Although there was a difference between groups (36.76 vs. 36.85), still this value is not considered in the threshold of fever.

Our study has its own limitations, which can be covered in the future studies. The open-label design of the study and relatively small patient population are the main limitations. Further randomized double-blind clinical trials with more patient population can be beneficial.

## Conclusion

In this study, we found that there were improvements in peripheral oxygen saturation and body temperature in both groups during the time of admission, but we did not find significantly better outcomes in the group who were treated with high-dose vitamin C in addition to the main treatment regimen at discharge.

## Data Availability

Not applicable.

## Abbreviations

HDIVC: High-dose intravenous vitamin C
ICU: Intensive care unit
COVID-19: The coronavirus disease 2019
SARS-COV-2: Severe acute respiratory syndrome coronavirus 2
IV: Intravenous
EBV: Epstein–Barr virus
ARDS: Acute respiratory distress syndrome
G6PD: Glucose-6-phosphate dehydrogenase
ESRD: End-stage renal disease
CBC: Complete blood count
CRP: C-reactive protein
ESR: Erythrocyte sedimentation rate
RR: Respiratory rate
HR: Heart rate
SpO_2_: Peripheral capillary oxygen saturations
IRB: Institutional review board
TUMS: Tehran University of Medical Sciences
ANOVA: Analysis of variance model
HSV-1: Herpes simplex virus type 1
TTIC: Time to clinical improvement
NEWS2: National Early Warning Score 2
IMVFD28: Invasive mechanical ventilation-free days in 28 days
LOVIT-COVID: Lessening Organ Dysfunction with Vitamin C-COVID
